# Lateral resolution of DGT LA-ICP-MS for chemical imaging of metal solutes

**DOI:** 10.1007/s00216-026-06500-7

**Published:** 2026-04-28

**Authors:** Gulnaz Mukhametzianova, Stefan Wagner, Thomas Prohaska

**Affiliations:** 1 Department of General, Analytical and Physical Chemistry, Chair of General and Analytical Chemistry, Montanuniversität Leoben, Franz Josef-Straße 18, 8700 Leoben, Austria; 2 Christian Doppler Laboratory for Inclusion Metallurgy in Advanced Steelmaking, Montanuniversität Leoben, Franz Josef-Straße 18, 8700 Leoben, Austria

**Keywords:** Metals, Passive sampling, Imaging, Diffusion, Mass spectrometry, Laser ablation

## Abstract

Diffusive gradients in thin films (DGT) coupled with laser ablation inductively coupled plasma mass spectrometry (LA-ICP-MS) is increasingly applied for high-resolution chemical imaging of metal solutes, yet its effective lateral resolution has not been quantitatively assessed. This study provides the first targeted investigation of the lateral resolution achievable with state-of-the-art DGT LA-ICP-MS using copper (Cu) as a model analyte in three complementary experimental designs that isolate diffusion-controlled, geometry-controlled, and material-controlled constraints: (i) Controlled solute transport through a polytetrafluoroethylene (PTFE) foil with 5–100 µm holes revealed differential time-dependent broadening of solute and matrix patterns and the transition from geometry-preserving to diffusion-dominated behaviour. Full width at half maximum/minimum (FWHM)-based analysis showed very small apparent lateral “imaging-blur” coefficients for DGT-bound metals (Cu/Zn ≈10^−15^–10^−13^ m^2^ s^−1^), i.e., several orders of magnitude lower than typical ionic diffusion in hydrogels (≈10^−10^ m^2^ s^−1^), consistent with rapid immobilization at the DGT binding phases and modest lateral within-gel diffusion. (ii) Direct gel contact with Cu metal grids enabled quantitative comparison of nominal bar/hole dimensions with DGT-derived solute patterns, demonstrating that features down to ~25 µm are detectable under optimal contact and laser ablation conditions. (iii) Application to printed circuit boards confirmed the method’s ability to reproduce sub-mm Cu features on technologically relevant materials. Across experiments, gel-phase lateral diffusion and imperfect gel-sample contact were the dominant factors limiting spatial accuracy. These results define the practical lateral resolution of DGT LA-ICP-MS and provide guidance for designing and interpreting high-resolution solute imaging experiments in environmental and materials science.

**Graphical abstract:**

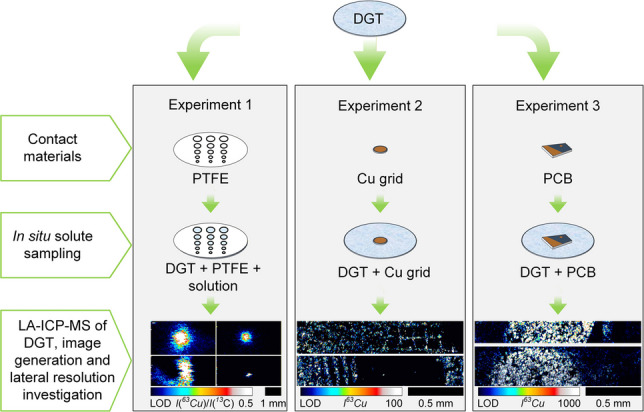

**Supplementary Information:**

The online version contains supplementary material available at 10.1007/s00216-026-06500-7.

## Introduction

Diffusive gradients in thin films coupled with laser ablation inductively coupled plasma mass spectrometry (DGT LA-ICP-MS) is a powerful chemical imaging technique for spatial analysis of labile metal solute species in heterogeneous systems [1]. Metal solutes are sampled in situ by a DGT gel consisting of a hydrogel matrix impregnated with analyte-selective binding phases, and the spatial distribution of the bound solute fraction is subsequently analysed by LA-ICP-MS. By combining time-integrated diffusive sampling with spatially resolved elemental analysis, the technique provides site-specific quantitative information on labile solute fluxes and concentration gradients, making it particularly valuable for environmental research [2–6]. Accordingly, DGT LA-ICP-MS has been widely applied to investigate biogeochemical metal cycling, as highlighted by recent applications in soils [7–9], sediments [10], and rhizospheres [11, 12]. Its application has been recently extended to materials science, where initial studies demonstrated its ability to resolve corrosive metal release and dynamic degradation processes in magnesium [13] and aluminium alloys [14], highlighting its potential for assessing interfacial reaction dynamics at solid-solution boundaries. An overview of published DGT LA-ICP-MS studies, including applied gel types, instrumentation, spatial resolution, and application fields, is provided in the Supplementary Information (Table S1).

Despite these advances, the spatial accuracy of DGT LA-ICP-MS remains a key challenge. Central to this is the technique’s lateral resolution, defined as the minimum distance at which two neighbouring features can be distinguished as separate entities [15]. Insufficient resolution results in geometric blurring and spatial averaging, which may obscure microscale reactivity hotspots and compromise interpretation of localized solubilization processes. Although environmental applications typically require only sub-mm resolution (> 100 µm) to resolve broader biogeochemical gradients [16], many materials-related solubilization processes occur at micrometer scales (< 100 µm) [13, 14], placing high demands on spatial accuracy.

The achievable lateral resolution of DGT LA-ICP-MS is governed by both laser ablation parameters and processes related to DGT sampling. On the instrumental side, laser spot size, fluence, scan speed, repetition rate, and spot overlap determine the smallest resolvable feature size, while aerosol transport and washout dynamics further influence image sharpness [17, 18]. These parameters are overall well understood and can be optimized using established protocols and modern instrumentation [17, 19, 20]. In contrast, DGT-specific processes control how solute distributions are captured before analysis. Among these, the particle size and homogeneity of the binding phase within the DGT gel [2, 21–23] and gel deformation during drying [14, 21, 24] have been investigated in detail and are well constrained. By comparison, two processes remain poorly quantified: (i) the quality and duration of gel-sample contact during deployment, and (ii) lateral diffusion of solutes within the hydrated binding gel matrix. Both processes are expected to exert a dominant influence on the effective lateral resolution of DGT LA-ICP-MS images, particularly for micrometer-scale imaging applications. However, they have not yet been systematically investigated under well-defined geometric boundary conditions.

Lateral diffusion in hydrogels refers to solute transport through the water-filled polymer network parallel to the gel surface [25, 26]. In the context of DGT sampling, solutes are not necessarily immobilized instantaneously upon arrival at the gel surface but may remain partially mobile until captured by the binding phase, allowing lateral redistribution within the gel. In imaging applications, this process constitutes an intrinsic blurring mechanism that is independent of the analytical resolution of the LA-ICP-MS system. Previous experimental and modelling studies have demonstrated that lateral diffusion within the diffusive hydrogel of conventional bulk DGT samplers can significantly affect measured solute fluxes, with reported increases of up to ~ 20% [27, 28]. However, these studies addressed corrections to integrated mass uptake and DGT concentration calculations and did not consider lateral diffusion within the binding gel or its impact on spatial accuracy. Consequently, the intrinsic spatial limits imposed by lateral diffusion in DGT LA-ICP-MS imaging remain essentially unconstrained by experimental data.

Beyond intrinsic gel diffusion, the effective lateral resolution is further influenced by the quality of gel-sample contact during deployment. Even minor deviations from ideal contact, such as micro gaps, surface roughness, or recessed features, can locally alter diffusion pathways and promote lateral solute redistribution before immobilization [14]. These effects become particularly important when imaging microstructures, where the characteristic feature size approaches the length scale over which lateral diffusion operates. Systematic evaluation of how well DGT preserves imposed geometries under direct contact conditions is therefore essential for assessing its applicability to materials with complex or heterogeneous surfaces.

The objective of this work is to provide the first systematic experimental assessment of the lateral resolution capabilities and limitations of DGT LA-ICP-MS, with specific emphasis on materials science applications. To progressively disentangle intrinsic gel diffusion from contact-related effects and to link controlled model systems to realistic materials, three complementary experiments were designed using copper (Cu) as a model analyte: (1) controlled solute diffusion through a patterned polytetrafluoroethylene (PTFE) foil to quantify intrinsic lateral diffusion within the gel under idealized boundary conditions; (2) direct deployment of DGT gels onto Cu grids with well-defined micrometer-scale geometries to assess the combined influence of lateral diffusion and gel-sample contact; and (3) application to printed circuit boards (PCBs) containing discrete Cu phases to evaluate lateral resolution under realistic, heterogeneous surface conditions.

## Materials and methods

### Laboratory procedures, solutions, and materials

Ultrapure water (σ = 0.055 µS cm^−1^) was obtained from a Milli-Q device (Milli-Q IQ 7000 system, Merck Millipore, Burlington, Massachusetts, USA) and used in all experiments. Nitric acid (HNO_3_, *w* = 65%, p.a.; Chem-Lab, Zedelgem, BE) was purified by sub-boiling in perfluoralkoxy-polymer (PFA) units (DST-4000, Savillex, Eden Prairie, Minnesota, USA). Plastic consumables used for DGT preparation, immersion, and analysis were soaked in HNO_3_ (*w* = 5–10%) for at least 24 h, rinsed with ultrapure water, and dried under laminar flow (Spetec GmbH, Erding, DE). DGT immersion solutions were prepared from Cu (γ = 1000 mg L^−1^ in 2% HNO_3_; Merck, Darmstadt, DE) and Zn (γ = 999 mg L^−1^ in 2% HNO_3_; Merck) single-element stock solutions. For pH adjustments, aqueous NaOH (*c* = 0.001 mol L^−1^) prepared from sodium hydroxide monohydrate (NaOH × 1H_2_O; ≥ 99.996%, metals basis, Thermo Scientific, Waltham, Massachusetts, USA) was used. Ethanol absolute (≥ 99.8%; VWR, Vienna, AT) was used for cleaning PCB surfaces. An analytical balance (BL224 BASIC, XS instruments, Carpi, IT) with a readability of 0.0003 g was used for weighing. Cleaning of consumables, preparations of DGT gels and immersion solutions, as well as ICP-MS analyses were performed in an ISO class 8 clean room. 

### DGT gel preparation

Polyurethane-Chelex-Zr(OH)₄ (PU-C-Zr) DGT-binding gels [14, 29] were used in all experiments. These gels were selected because they combine high mechanical stability, minimal shrinkage, spatially homogeneous binding-phase distribution, and rapid analyte immobilization, i.e., properties that are critical for high-resolution DGT imaging applications. Previous work demonstrated that polyurethane-based gels provide superior preservation of localized solute patterns at the microscale [14], owing to their uniform polymer network with lower water contents and reduced deformation upon drying compared to conventional polyacrylamide-based gels. Moreover, PU-C-Zr gels have been extensively characterized and applied in high-resolution DGT LA-ICP-MS studies (Table S1), making them a relevant choice for evaluating the technique’s lateral resolution capabilities. Synthesis of PU-C-Zr gels was conducted as described in Mukhametzianova et al. [14].

### Conceptual experimental design

The experimental design was structured to isolate and quantify two key DGT-specific processes governing spatial resolution: (i) lateral diffusion within the gel and (ii) gel-sample contact quality. To disentangle these effects, three complementary experiments were performed. Experiment 1 targeted intrinsic lateral diffusion under well-defined geometric boundary conditions using a patterned PTFE foil with micrometer-scale openings. This configuration decouples diffusion from surface-related artefacts and enables time-resolved assessment of lateral spreading. Experiments 2 and 3 investigated spatial accuracy under direct gel-sample contact. In Experiment 2, Cu grids with defined one- and two-dimensional geometries were used to evaluate how well DGT LA-ICP-MS reproduces imposed microstructures under controlled contact conditions. Experiment 3 extended this approach to PCBs, representing more complex and heterogeneous materials. Copper was used as a model analyte in all experiments due to its high binding affinity to PU-C-Zr gels [14, 29, 44], which promotes rapid immobilization and thereby limits lateral diffusive spreading, as well as its high technological relevance in materials systems. An overview of the three experimental approaches is provided in Table 1.

**Table 1:** Summary of the experimental conditions (details on contact materials, contact times, and immersion solutions are provided in the following sections)

Experiment	DGT gel	Contact material	Contact time	Immersion solution
1	PU-C-Zr	PTFE foil	6 h, 24 h	Yes
2	PU-C-Zr	Cu grid	5–20 min	No
3	PU-C-Zr	PCB	15 min	No

### Experiment 1: solute diffusion restriction using PTFE foil

**Fig. 1:**
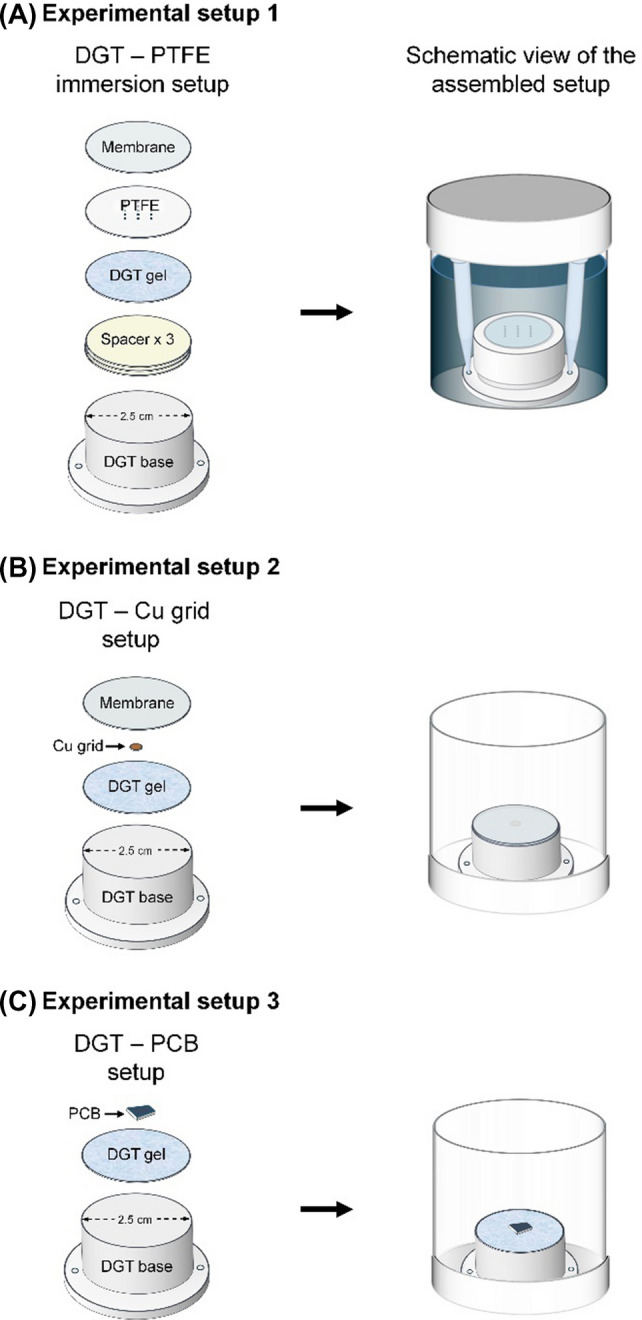
Schematic representation (not to scale) of the three DGT-based experimental setups used in this study, including (**A**) piston-type DGT-PTFE immersion setup for diffusion-restricted uptake, (**B**) DGT-Cu grid contact setup, and (**C**) DGT-PCB contact setup

A piston-type immersion setup (Fig. 1A) with a DGT gel overlain by a patterned PTFE foil was used to establish well-defined geometric boundary conditions for solute uptake. In addition to Cu, zinc (Zn) was used as a model analyte in this experiment to investigate differences in spatial adsorption for analytes with high (Cu) and moderate (Zn) binding affinity on PU-C-Zr gels [14, 44].

**Fig. 2:**
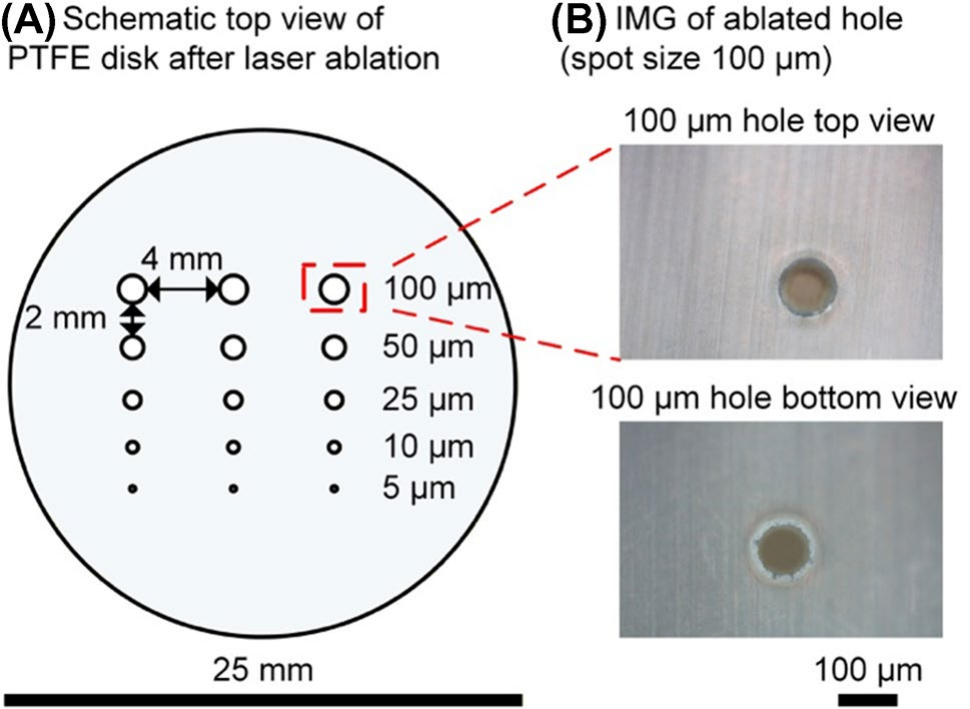
Schematic top view (not to scale) of the PTFE foil disk after laser ablation of 15 holes with nominal diameters of 100 µm, 50 µm, 25 µm, 10 µm and 5 µm (**A**), and optical micrographs of a representative 100 µm ablated hole (**B**)

The original PTFE foil sheet (thickness = 25 µm; Hightechflon GmbH, Konstanz, DE) was cut to disks (diameter = 25 mm) and laser-ablated to produce circular openings (i.e., holes) with nominal diameters of 100, 50, 25, 10, and 5 µm (three each; Fig. 2A; ablation parameters are provided in Table S2). The PTFE disks were acid-cleaned by soaking in 30 mL of HNO_3_ (*w* = 10%) for 24 h, rinsed with ultrapure water, followed by an additional 24 h wash in 30 mL of ultrapure water to ensure thorough cleaning of the ablated openings, and then dried. Hole geometry and potential debris were assessed by optical microscopy (StereoDiscovery V12/V20 and AxioImager M1, both, Carl Zeiss Microimaging GmbH, Jena, DE). The cleaned PTFE disk was placed with its debris-free side facing the PU-C-Zr gel (top side of the foil; Fig. 2B). The gel-foil assembly was placed on a stack of three plastic spacers (diameter = 25 mm; thickness = 0.4 mm) above the DGT base, overlaid with a polyethersulfone (PES) membrane (diameter = 25 mm; thickness = 0.14 mm; pore size = 0.45 µm; Sartorius, Göttingen, DE), and secured using a DGT sampler cap (exposure window diameter = 20 mm). The assembly was placed in a plastic vessel, immobilized at the bottom using two 1 mL pipette tips, and immersed in 100 mL of solution containing Cu (γ = 100 µg L^−1^) and Zn (γ = 100 µg L^−1^). Bromothymol blue (BTB) was added to the immersion solution to visualize exposed gel regions and assess potential lateral spreading of the solution matrix on the gel surface. For this purpose, 100 mg of BTB was dissolved in 50 mL ultrapure water, and 5 mL of this solution was added to the immersion solution, resulting in a dark green colour. The vessel was sealed and agitated on a horizontal shaker at 70 rpm for either 6 h or 24 h. The pH of the immersion solution was measured before and after the experiment and remained stable at 6.34 ± 0.04.

Despite the green solution, the BTB-stained regions appeared yellow/orange in wet conditions and red after drying the gel, reflecting adsorption-induced spectral shifts of BTB on the PU-C-Zr matrix, which locally stabilizes the protonated dye form independent of the bulk solution pH [30]. Importantly, drying did not alter the lateral extent of the stained regions.

After deployment, the assemblies were disassembled, and the gels were dried and mounted on glass plates for LA-ICP-MS analysis as described previously [6]. The stained regions on the gel surface were visualized by optical microscopy. Two additional gels served as blanks and were stored in ultrapure water without exposure to the immersion solution. A photographic overview of the setup is provided in the Supplementary Information (Fig. S1A).

### Experiment 2: geometrically defined diffusion using Cu grids

A contact-based setup (Fig. 1B) was used to evaluate spatial resolution under direct gel-sample interaction. To prevent drying of the PU-C-Zr gel in the absence of an immersion solution, a humid environment was established by placing a cleanroom tissue moistened with ultrapure water in a vessel lid. The DGT base was positioned on the moist tissue and a PU-C-Zr gel was placed onto it. Two commercially available Cu grids were used as model structures: a parallel-bar grid (G400PB) and a thin-bar variable mesh grid (Cu TVM) (Gilder Grids Ltd., Lincolnshire, UK; purchased from Electron Microscopy Science, Hatfield, Pennsylvania, USA). Their geometric properties are summarized in Table 2. Prior to use, the grids were stored in their original vials at room temperature and imaged by optical microscopy to visualize their structural dimensions.

**Table 2:** Geometrical properties of Cu grids ("n/a"–not applicable)

Cu grid	Diameter (mm)	Hole (µm)	Bar (µm)	Mesh
G400PB	3.05	22	40	n/a
Cu TVM	3.05	125	40	150
		90	35	200
		58	25	300
		37	25	400

The grids were placed directly onto the gel surface and covered with a PES membrane to ensure uniform contact as good as possible. The grid position was marked on the gel using a text marker to enable spatial referencing during LA-ICP-MS analysis. The vessel was sealed and kept at room temperature for 5–20 min. This time window was selected based on preliminary tests using a Cu wire, which indicated rapid analyte accumulation at the gel interface, with shorter times yielding insufficient signal and longer times causing local saturation and loss of spatial contrast. After deployment, the membrane and grids were removed, and the gels were dried and mounted on glass plates for analysis. An additional PU-C-Zr gel served as a blank and was kept in ultrapure water throughout the experiment. A photographic overview of the setup is provided in the Supplementary Information (Fig. S1B).

### Experiment 3: DGT deployment on PCBs with defined Cu phases

PU-C-Zr gels were deployed on PCBs using the same contact-based setup (Fig. 1C) and handling procedures described for Experiment 2 (see ”). In contrast to Cu grids, PCBs represent a more complex and heterogeneous Cu source with defined Cu phases embedded in a polymer matrix. PCB samples were obtained from a batch of electrical and electronic equipment waste (audio and visual appliances, personal care products, culinary equipment, etc.) supplied by Envie 2E Midi-Pyrénées (Portet-sur-Garonne, FR). Two PCB samples were prepared by manually dismantling the products and cutting regions containing Cu features of interest. Prior to deployment, PCB surfaces were cleaned with ethanol absolute and ultrapure water, dried, and imaged by optical microscopy. The PCBs were placed directly onto the PU-C-Zr gel surface, and their positions were marked for spatial referencing. The assemblies were sealed inside a vessel and kept at room temperature for 15 min. Blank gels were prepared as described above. After deployment, the PCBs were removed, and the gels were dried and mounted on glass plates for analysis. A photographic overview of the setup is provided in the Supplementary Information (Fig. S1C, D).

### LA-ICP-MS analysis

The analyte distribution on the dried PU-C-Zr gels was investigated using a nanosecond imageGEO 193 nm ArF excimer laser ablation system (Elemental Scientific Lasers, Bozeman, Montana, USA) coupled to a quadrupole ICP-MS (NexION 2000B, Perkin Elmer, Waltham, Massachusetts, USA). The gels were analyzed using an imaging XYR line scan mode, with measurement durations ranging from 12 min to 4.7 h per sample. Helium was applied as carrier gas at a flow rate of 0.8 L min^−1^ to transport the aerosol, and the Dual-Concentric Injector (DCI) system (Elemental Scientific Lasers) was employed to combine Helium with Argon gas before introduction into the ICP-MS. The signal intensities of ^13^C, ^63^Cu, ^65^Cu, and ^66^Zn were recorded as counts during the analytical runs. Prior to sample analysis, at least three ablation lines were performed on each gel blank, with line lengths ranging from 1 to 4 mm. Furthermore, a gas blank was recorded for up to 10 s before the start and after the completion of each line scan to monitor the analyte background. The washout times for ^63^Cu, ^65^Cu and ^66^Zn were below 91 ms, while the washout time for ^13^C was approximately 100 ms.  LA-ICP-MS parameters for all analyses are provided in the Supplementary Information (Table S3).

For Experiment 1 (LA-ICP-MS spot size 50 µm), the resulting gas blank-corrected signal data points were normalized using ^13^C as an internal standard [5, 9]. However, normalization was not applied in Experiments 2 and 3 (LA-ICP-MS spot size 5 µm). To justify this decision, the ^13^C signal obtained during ablation of the region of interest (ROI) on the gel in Experiment 1 (6 h exposure; ROI 100 µm center) was compared with the ^13^C signal from an additional control experiment using a calibration standard DGT gel with a homogeneous Cu distribution. The ROI was ablated using a 50 µm spot size, corresponding to the laser ablation parameters in Experiment 1, whereas the standard gel was ablated with a 5 µm spot size, matching the conditions of Experiments 2 and 3. The results are summarized in Fig. S2. During LA-ICP-MS ablation with a 50 µm spot size, the ^13^C signal was clearly above background and remained highly stable, decreasing by only 3.2% over 40 consecutive lines (Fig. S2A1, A2). In contrast, ablation with a 5 µm spot size produced ^13^C intensities that did not exceed the background level (Fig. S2B1, B2). These results indicate that ^13^C cannot be reliably used as an internal normalization standard at a 5 µm spot size under the experimental conditions.

### Data evaluation and image analysis

Data evaluation was performed using Microsoft Excel (Microsoft Corporation, Redmond, Washington, USA). Gas blank correction was applied by subtracting the average gas blank signal intensity for each analyte. For Experiment 1, gas blank-corrected signals were normalized to ^13^C as an internal standard [13, 29]. To enhance signal consistency and minimize the impact of signal spikes on subsequent analyses, outlier correction was applied to gel blank signals following the procedure of Mukhametzianova et al. [14].

Color-coded images were generated using Fiji ImageJ (National Institute of Health, Bethesda, Maryland, USA) [31], with each pixel representing a single data point. Images were scaled without pixel interpolation, exported as TIFF files, and assembled into final figures using Microsoft Visio (Microsoft Corporation). Fiji ImageJ was also used for 2D profile extraction, with profile data exported as .csv files for further processing in Microsoft Excel.

For Experiment 1, extracted 2D profiles were used to calculate lateral diffusion coefficients and to estimate diffusion diameters. For solute images, peak baselines and maxima were determined, whereas for microscopic images, baselines and minima were determined. In both cases, the half-maximum or half-minimum positions were calculated, and the corresponding full width at half maximum or minimum (FWHM) was measured. The lateral diffusion coefficient was calculated from the temporal increase in peak width assuming Gaussian broadening according to Eq. 1:

1 $${D}_{\mathrm{lat}}=\frac{[\mathrm{FWHM}{\left({t}_{2}\right)}^{2}-\mathrm{FWHM}{\left({t}_{1}\right)}^{2}]}{[16\mathrm{ln}2\left({t}_{2}-{t}_{1}\right)]}$$

where *D*_lat_ (m^2^ s^−1^) is the lateral diffusion coefficient, FWHM (m) is the full width at half maximum or minimum, and *t* (s) is time. Diffusion diameters were estimated using a threshold defined as the average baseline signal + 10 × standard deviation (SD).

For Experiments 2 and 3, 2D profiles were evaluated to assess spatial accuracy at the gel-sample interface. In Experiment 2, profiles were extracted from direct LA-ICP-MS images of Cu grids and from the corresponding Cu solute images, with three profiles per image (*n* = 3) measured across grid bars and openings to determine feature widths, average values, and standard deviations. In Experiment 3, analysis was limited to Cu solute images and corresponding microscopic images of PCB surfaces.

## Results and discussion

### Experiment 1: lateral resolution assessment via solute diffusion restriction

**Fig. 3:**
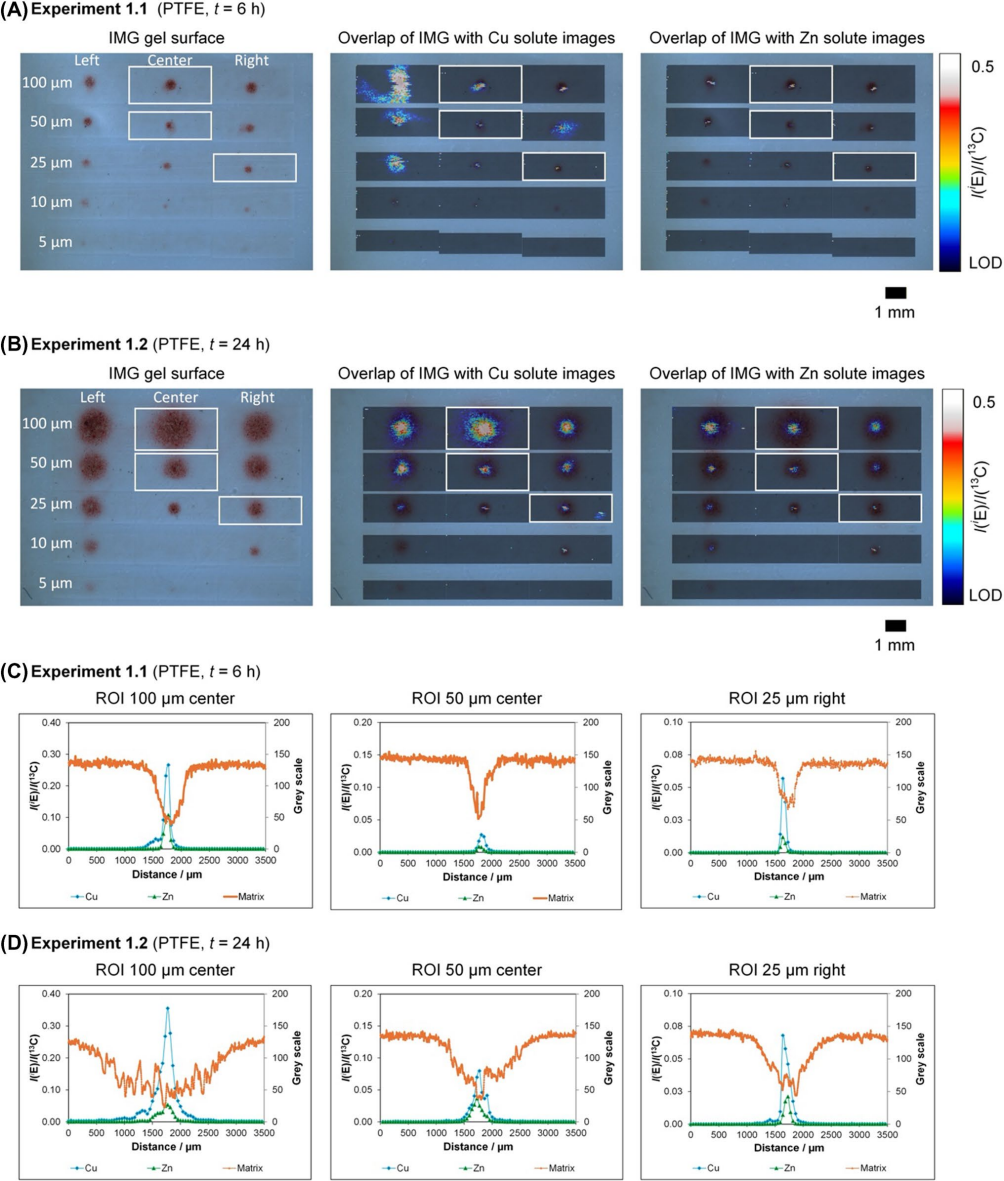
Microscopic images of PU-C-Zr gels and corresponding Cu and Zn solute images after 6 h (**A**) and 24 h (**B**) exposure to the immersion solution using the patterned PTFE foil setup, followed by LA-ICP-MS analysis. Semi-transparent (50%) solute images for ROIs with nominal diameters of 100 µm, 50 µm, 25 µm, 10 µm, and 5 µm are overlaid on the red-stained regions on the gel surface, which mark the positions of the corresponding PTFE holes. Panels (**C**) and (**D**) show representative 2D line profiles extracted from selected ROIs (100 µm center, 50 µm center, and 25 µm right) after 6 h and 24 h exposure for both analytes, respectively. The *y*-axis represents blank-corrected and ^13^C-normalized signal intensities (*I*) of the isotopes (^i^*E*) ^63^Cu and ^66^Zn

Experiment 1 evaluated how DGT LA-ICP-MS reproduces geometrically defined diffusion pathways and how deployment time influences lateral mass transport within the gel. Solute distributions captured by DGT sampling and LA-ICP-MS imaging (Cu, Zn) were directly compared with matrix transport visualized by bromothymol blue (BTB) staining and optical microscopy after 6 h and 24 h immersion. This approach enabled a time-resolved assessment of lateral diffusion under well-defined boundary conditions and provided a quantitative basis for evaluating how effectively DGT LA-ICP-MS preserves the imposed diffusion geometries. Gel micrographs after deployment, resulting Cu and Zn solute images, and representative 2D profiles are shown in Fig. 3, while lateral diffusion coefficients (*D*_lat_) derived from time-dependent FWHM broadening are presented in Table 3.

**Table 3:** Summary of full width at half maximum/minimum (FWHM) values for solute images and microscopic gel surface images with corresponding diffusion coefficients (“x” indicates that the analyte signal was below the limit of detection for solute images or did not notably exceed the background level for microscopic images, “n.e.”—non evaluable)

PTFE hole size (µm)	PTFE hole location	Cu FWHM (µm)		*D*_lat_ (m^2^ s^−1^)	Zn FWHM (µm)		*D*_lat_ (m^2^ s^−1^)	IMG gel surface FWHM (µm)		***D***_lat_ (m^2^ s^−1^)
		6 h	24 h		6 h	24 h		6 h	24 h	
100	Left	220	270	3.4 × 10^−14^	120	400	2.0 × 10^−13^	500	1380	2.3 × 10^−12^
100	Center	110	205	4.2 × 10^−14^	110	290	1.0 × 10^−13^	470	1700	3.7 × 10^−12^
100	Right	140	300	9.8 × 10^−14^	140	320	1.2 × 10^−13^	510	1240	1.8 × 10^−12^
50	Left	120	170	2.0 × 10^−14^	110	170	2.3 × 10^−14^	350	990	1.2 × 10^−12^
50	Center	140	250	6.0 × 10^−14^	120	190	3.0 × 10^−14^	200	1050	1.5 × 10^−12^
50	Right	110	240	6.3 × 10^−14^	100	140	1.3 × 10^−14^	350	1160	1.7 × 10^−12^
25	Left	220	290	5.0 × 10^−14^	n.e.	n.e.	n.e.	230	720	6.5 × 10^−13^
25	Center	70	80	2.1 × 10^−15^	90	100	2.6 × 10^−15^	290	475	2.0 × 10^−13^
25	Right	90	170	2.9 × 10^−14^	90	120	8.8 × 10^−15^	270	680	5.4 × 10^−13^
10	Left	90	x	x	90	310	1.2 × 10^−13^	300	500	2.2 × 10^−13^
10	Center	100	x	x	100	x	x	80	x	x
10	Right	110	110	0	x	100	x	140	410	2.1 × 10^−13^
5	Left	125	x	x	110	190	3.3 × 10^−14^	160	190	1.5 × 10^−14^
5	Center	x	x	x	x	280	x	x	x	x
5	Right	x	x	x	x	x	x	x	x	x

After 6 h, BTB-stained regions on the gels largely matched the imposed PTFE hole geometry, although slight asymmetries indicated localized microgaps at the PTFE-gel interface (Fig. 3A). After 24 h, staining extended well beyond the nominal hole diameters, consistent with progressive lateral diffusion of the immersion solution along the gel surface (Fig. 3B). Cu and Zn images reproduced the overall geometry of the PTFE holes but showed substantially smaller lateral extents than the stained regions, consistent with rapid cation adsorption by the gel (Fig. 3A, B). Quantitative profiles for 100 µm holes (Fig. 3C, D) indicated apparent diffusion widths of ~ 600 µm (Cu) and ~ 250 µm (Zn) after 6 h, increasing to ~ 1380 µm (Cu) and ~ 550 µm (Zn) after 24 h, whereas matrix diffusion increased from ~ 900 to ~ 2450 µm, consistent with the √t dependence expected for Fickian diffusion [32]. In some cases, Cu distributions extended beyond the stained boundaries (Fig.  3A, 100 µm, left), indicating microgaps that locally trapped solution and provided additional solute access.

For the smallest PTFE hole sizes (10 µm and 5 µm), Cu and Zn signals remained below their respective LODs despite clearly visible matrix staining (Fig. 3A, B). This absence of detectable analyte accumulation likely reflects a combination of factors. First, diffusive mass transfer through openings ≤ 10 µm is inherently limited by the small effective area (Δ*m*/Δ*t* ∝ *DA*Δ*c*/Δ*x*) [33, 34], resulting in pg-fg-level uptake that approaches the instrumental LOD of the LA-ICP-MS setup [14, 35]. Second, background analyte levels in the gels can obscure such low accumulated masses via reduced signal-to-background ratios [36–38]. Third, edge effects at the PTFE-gel boundary, including charge- or interface-mediated ion retardation, may locally reduce the effective concentration gradient driving diffusion [39, 40], an influence that becomes increasingly significant at very small feature sizes.

The *D*_lat_ values derived from FWHM broadening were very small for Cu and Zn, ranging from 2.1 × 10^−15^ m^2^ s^−1^ to 9.8 × 10^−14^ m^2^ s^−1^ for Cu and 2.6 × 10^−15^ m^2^ s^−1^ to 2.0 × 10^−13^ m^2^ s^−1^ for Zn (Table 3). In contrast, BTB matrix-related features exhibited consistently higher *D*_lat_ values, typically in the 10^−12^ m^2^ s^−1^ range. When the lateral diffusion was quantified using a conservative threshold-based diameter (baseline + 10 × SD) instead of the FWHM approach, *D*_lat_ values increased systematically (typically ~ 10^−13^ to 10^−12^ m^2^ s^−1^ for Cu/Zn; up to ~ 10^−12^ to 10^−11^ m^2^ s^−1^ for matrix/surface features). FWHM was finally preferred because it provides a threshold-independent and reproducible descriptor of lateral broadening that is consistent with Gaussian distributions expected for diffusion-driven blurring in hydrogels [25]. As it is defined at 50% of the peak height, FWHM is comparatively insensitive to baseline drift, spatial background heterogeneity, and low-intensity tails, yielding more robust between-image and between-element comparability. In contrast, the threshold-based diameter metric is highly sensitive to noise level, ROI selection, and subtle artefacts (e.g., weak halos or surface-related smearing), which can systematically inflate apparent diffusion extents and therefore overestimate lateral imaging uncertainty.

Interpreted as an imaging uncertainty term rather than a molecular diffusion coefficient, the very small FWHM-derived *D*_lat_ values obtained for Cu and Zn are consistent with rapid analyte immobilization at the gel’s binding sites, effectively suppressing lateral diffusion once ions reach the gel. It is therefore expected and indeed desirable that these apparent *D*_lat_ values are several orders of magnitude smaller than diffusion coefficients reported for metal ions in different hydrogels (~ 10^−10^ m^2^ s^−1^) [25, 41–43]. Matrix-related features derived from BTB staining exhibited larger *D*_lat_ values than Cu and Zn, consistent with the largely non-binding nature of the dye, which allows continued lateral spreading along the gel surface. Nevertheless, these matrix-derived *D*_lat_ values also remain well below the diffusion coefficients expected for freely diffusing species in highly hydrated hydrogels, likely reflecting the reduced water content and hence lower diffusivity of polyurethane-based PU-C-Zr gels (*w*_H2O_ = 50%) compared to pure polyacrylamide hydrogels (*w*_H2O_ = 95%) typically used in DGT [14, 42]. The generally larger *D*_lat_ values observed for Zn compared to Cu further illustrate that this “imaging-blur” metric integrates both lateral mobility and immobilization kinetics. Cu binds faster and more strongly to the Chelex resin in the PU-C-Zr gel [44], resulting in sharper spatial patterns, whereas Zn can diffuse slightly further over time. Together, these geometry-defined and time-resolved results demonstrate that lateral diffusion within the gel increases predictably with deployment time and progressively degrades spatial accuracy of DGT LA-ICP-MS imaging under otherwise well-controlled conditions.

### Experiment 2: impact of sample geometry on lateral resolution

**Fig. 4:**
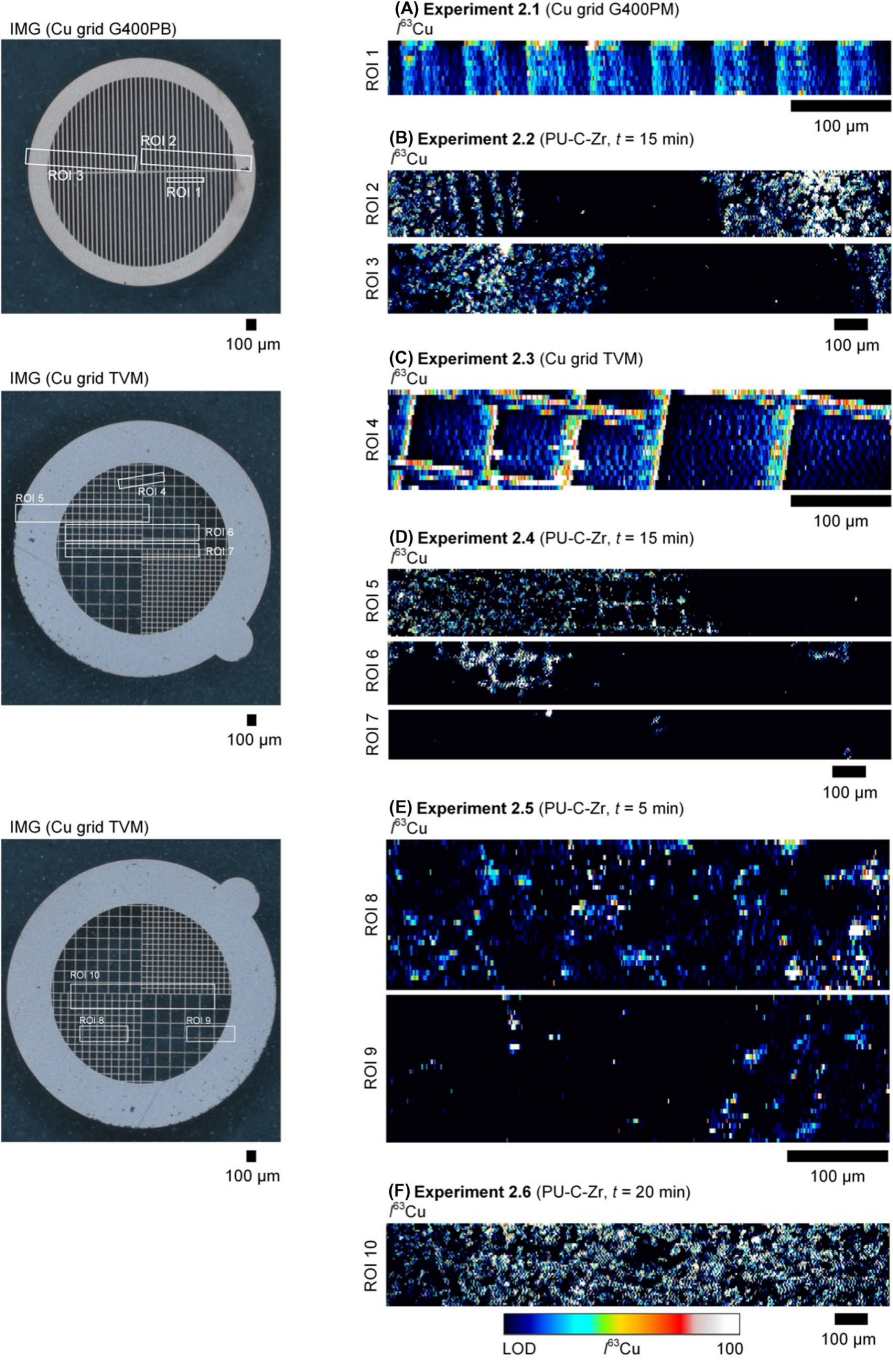
Microscopic images of G400PB and Cu TVM grids and corresponding Cu reference and solute images, obtained from LA-ICP-MS of (**A**) G400PB, (**B**) PU-C-Zr gel after 15 min contact with G400PB, (**C**) Cu TVM, (**D**) PU-C-Zr gel after 15 min contact with Cu TVM, (**E**) PU-C-Zr gel after 5 min contact with Cu TVM, and (**F**) PU-C-Zr gel after 20 min contact with Cu TVM

Experiment 2 evaluated the resolution limits achievable under direct gel-sample contact using two Cu grids (G400PB and TVM) with well-defined geometries (Fig. 4). Direct LA-ICP-MS ablation of the grids served as a geometric reference and confirmed that a 5 µm spot size accurately reproduced bar and hole dimensions in close agreement with manufacturer specifications (Table 2). The laser ablation parameters for grid ablation were matched to those used for gel ablation (except for energy), allowing to define the best-case image fidelity attainable under optimal analytical conditions and to establish the geometric accuracy against which DGT-derived patterns can be compared.

Figure 4 shows the direct LA-ICP-MS reference images of G400PB (Fig. 4A, ROI 1) and the 300-mesh region of Cu TVM (Fig. 4C, ROI 4), together with the corresponding Cu solute images obtained from PU-C-Zr gels after 15 min contact (Fig. 4B, ROIs 2–3; Fig. 4D, ROIs 5–7). In the reference image of G400PB, the measured bar and hole sizes (40.3 µm ± 2.3 µm, *n* = 3, and 24.3 µm ± 1.4 µm, *n* = 3) closely matched the nominal 40 µm and 22 µm values, demonstrating that the 5 µm spot size reliably resolves this parallel-bar pattern. The corresponding DGT solute images revealed only partial reproduction of the pattern: bars appeared narrower (24.6 µm ± 8.8 µm,* n* = 3) and holes broader (39.7 µm ± 6.8 µm,* n* = 3), reflecting modest lateral diffusion at the DGT-grid contact and/or small variations in DGT-grid contact.

A comparable outcome was observed for the TVM grid. The reference image of the 300-mesh region produced clear square patterns with bar and hole sizes of 25.2 µm ± 0.5 µm (*n* = 3) and 61.6 µm ± 3.7 µm (*n* = 3) respectively, consistent with nominal values of 25 µm and 58 µm. After 15 min DGT-grid contact, the solute images again showed partial pattern preservation: ROI 5 displayed bars of 16.4 µm ± 4.2 µm (*n* = 3) and holes of 64.9 µm ± 8.6 µm (*n* = 3), ROI 6 showed similar bar and hole sizes (17.6 µm ± 4.5 µm,* n* = 3, and 68.3 µm ± 6.4 µm, *n* = 3), while ROI 7 exhibited no discernible features. As with G400PB, the DGT-derived bars were systematically narrower and the holes broader than in the reference images, quantifying the degree of diffusion-induced broadening even under short exposure conditions.

The incomplete reproduction of the narrow bars in both grids is most plausibly attributed to slight non-uniformities in gel-grid contact as well as heterogeneous Cu release across the grid surface. In ROI 2, good contact is evident along the left side and at the perimeter Cu ring, but the pattern disappears in the central region where contact was likely lost. ROI 3 shows a similar trend: the initial portion of the grid is well defined on the left, whereas the pattern deteriorates progressively toward the right. Comparable contact-related inconsistencies are observed in ROIs 6 and 7. Despite these local variations, the results clearly demonstrate that DGT LA-ICP-MS can resolve µm-scale spatial patterns in both 1D (parallel bar) and 2D (square mesh) geometries when using a laser spot size of 5 µm.

To assess the influence of deployment time, additional experiments with the TVM grid were conducted using 5 min and 20 min contact (Fig. 4E, F, ROIs 8–10). After 5 min, Cu accumulation was detectable but insufficient to resolve the square pattern, whereas 20 min contact led to overaccumulation that obscured structural details. These observations confirm that the 15 min deployment time used in the main experiment represents an optimal balance between sufficient analyte uptake and preservation of geometric contrast. Collectively, the results indicate that features down to ~ 25 µm can be reliably resolved, provided that gel-sample contact is uniform and contact time is carefully controlled.

### Experiment 3: lateral resolution assessment in PCB analysis

**Fig. 5:**
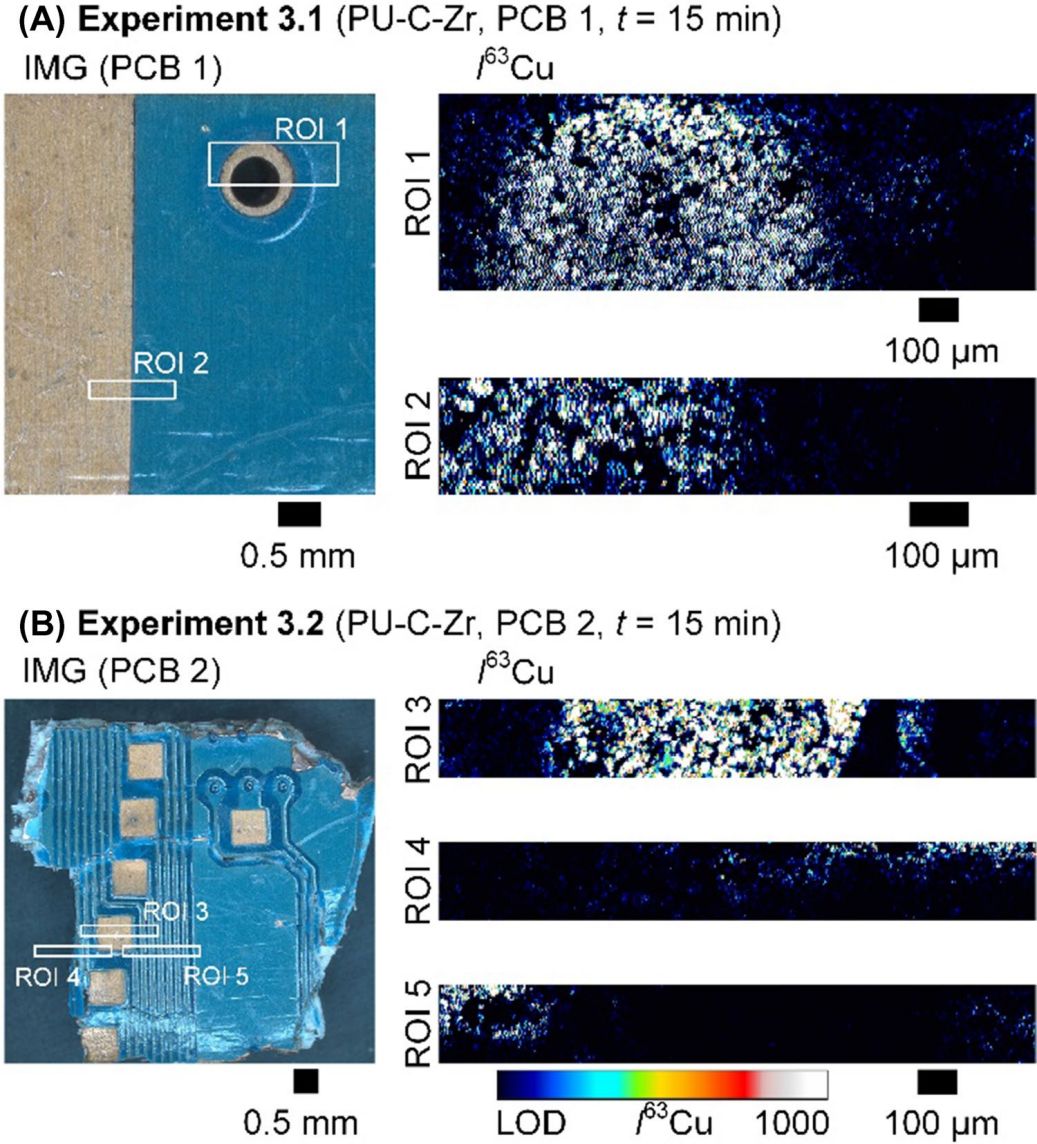
Microscopic images of PCB 1 and PCB 2 showing metallic Cu regions (brown), polymer regions (blue), and the central hole (black) within the circular Cu feature on PCB 1, alongside the corresponding Cu solute images obtained from PU-C-Zr gels after 15 min of gel-PCB contact. **A** ROIs 1 and 2 reproduce the circular and square Cu features on PCB 1. **B** ROIs 3–5 show the sub-millimeter Cu square features on PCB 2, with minor waviness attributed to slight gel-PCB contact variations or irregular corrosion

Experiment 3 extended the contact-based approach established in Experiment 2 to a more complex and heterogeneous substrate, namely printed circuit boards (PCBs) containing Cu features embedded in a polymer matrix (Fig. 5). Using the same deployment time (15 min) identified as optimal for preserving geometric contrast in Experiment 2, PU-C-Zr gels were brought into direct contact with two PCB samples to evaluate how well DGT LA-ICP-MS reproduces Cu feature geometry under realistic surface conditions.

The resulting Cu solute images closely reflected the overall geometry and dimensions of the underlying PCB features. For PCB 1 (Fig. 5A), ROIs 1 and 2 correspond to circular and square Cu features, respectively. The circular feature exhibited a lateral extent of ~ 960 µm in both the Cu solute image and the corresponding microscopic PCB image, with a sharp boundary between the Cu and polymer phases. This agreement demonstrates that, when good gel-sample contact is maintained, DGT LA-ICP-MS can faithfully reproduce sub-mm Cu features on complex substrates.

A notable exception is the central hole within the circular Cu region, which was not resolved in the solute image (Fig. 5A, ROI 1). As in Experiment 2, this reflects a contact-related limitation: the gel bridges across recessed features, allowing Cu released within the cavity to be scavenged directly by the gel and thereby masking the true geometry. This observation reinforces that recessed or non-planar features may remain unresolved if direct gel-sample contact is not ensured.

Cu solute images obtained from PCB 2 (Fig. 5B, ROIs 3–5) similarly reproduced the size and shape of square Cu features. Minor edge waviness observed in ROIs 4 and 5 likely arises from small variations in gel-sample contact or spatially heterogeneous corrosion of the PCB surface, analogous to the contact-related artefacts observed for the Cu grids in Experiment 2. In ROI 3, the Cu feature length measured ~ 670 µm in the solute image compared with ~ 700 µm in the microscopic image, corresponding to agreement within approximately ± 5%.

Overall, the PCB experiments corroborate the findings of Experiment 2 under more realistic conditions: lateral resolution is primarily governed by gel-sample contact quality and deployment time, rather than by analytical limitations of the LA-ICP-MS system. While fine µm-scale features require uniform, planar contact to be resolved, sub-mm Cu structures on heterogeneous substrates can be captured with good dimensional accuracy, confirming the applicability of DGT LA-ICP-MS for spatially resolved metal release studies on real-world materials.

### Capabilities and limitations of DGT LA-ICP-MS for high-resolution imaging

The combined results from Experiments 1–3 provide a systematic, geometry-controlled assessment of the practical lateral resolution of DGT LA-ICP-MS imaging. Under optimal conditions, a lateral resolution of ~ 25 µm is achieved. This resolution is constrained primarily by DGT-specific processes, whereas LA-ICP-MS parameters play a minor role once optimized.

Geometry-defined diffusion experiments (Experiment 1) demonstrate that lateral diffusion within the hydrated gel constitutes the dominant intrinsic limitation to resolution. Solute mass arriving at the gel surface spreads laterally over time, producing diffusion halos that increase predictably with deployment duration. Although rapid immobilization by the DGT binding phases strongly suppresses lateral diffusion compared to non-binding matrix tracers, it does not fully confine it. The resulting apparent lateral diffusion coefficients therefore represent imaging-uncertainty terms rather than molecular diffusion constants, capturing the combined effects of transport and binding on spatial accuracy. These findings quantitatively complement earlier models [45] by providing direct experimental evidence under imaging-relevant conditions.

The magnitude of lateral diffusion observed in this study must be interpreted in the context of the used polyurethane-based binding gels. These gels differ fundamentally from conventional polyacrylamide-based hydrogels in polymer structure and water content [14], which is expected to reduce effective diffusivity relative to highly hydrated gels. Consequently, lateral diffusion inferred from modelling and experimental observations on pure polyacrylamide hydrogels [27, 28] cannot be transferred directly to polyurethane gels. The lack of systematic diffusion data for polyurethane gels represents a current knowledge gap, and targeted diffusion cell experiments will be required to establish gel-specific diffusivity and its effects on lateral resolution in DGT-based imaging. Accordingly, the practical lateral resolution established in this study is specific to the PU-C-Zr system and should not be transferred directly to other DGT gel types without accounting for differences in gel structure, water content, binding-phase distribution, deformation behaviour, and analyte immobilization kinetics.

Across all experiments, deployment time emerges as a primary and experimentally controllable determinant of spatial accuracy. Short deployments preserve the geometry of solute release with high fidelity, whereas prolonged deployments lead to progressive lateral diffusion that decouples measured solute distributions from the underlying microstructure. In Experiment 1, sharp diffusion boundaries were preserved only at short exposure times, while longer deployments yielded broadened solute patterns that no longer reflected the imposed geometry. Experiments 2 and 3 further showed that, even when LA-ICP-MS resolves fine structures with high analytical precision, extended DGT deployments result in solute images that represent time-integrated fluxes rather than instantaneous spatial heterogeneity. These findings confirm previous DGT LA-ICP-MS studies showing that deployment time is a key tuning parameter that must be optimized according to feature size and analyte flux [13, 14].

A second major limitation arises from non-uniform gel-sample contact, which locally alters solute access pathways and produces spatial artefacts independent of diffusion kinetics. Microgaps at the gel interface facilitated unintended lateral solution trapping in Experiment 1, while incomplete reproduction of narrow Cu grid features and localized discontinuities in Experiments 2 and 3 point to subtle contact imperfections or heterogeneous surface reactivity, consistent with previous corrosion-related DGT studies [13, 14]. In the PCB experiments, recessed features such as drilled holes were systematically absent from DGT images, demonstrating that physical non-contact renders even geometrically dominant structures invisible. These observations highlight a fundamental aspect of DGT imaging: the method records the geometry of solute flux into the gel rather than the physical geometry of the substrate itself [45]. Consequently, microtopography, corrosion behaviour, and mechanical compliance strongly influence spatial accuracy, making uniform and intimate gel-sample contact essential for reproducible high-resolution imaging.

Once DGT-related constraints are minimized, LA-ICP-MS performance becomes enabling rather than limiting. Direct ablation of Cu grids confirmed that a 5 µm laser spot size yields geometrically accurate reference images for features down to a few tens of micrometers, consistent with established LA-ICP-MS imaging capabilities [19, 20]. Experiments 2 and 3 showed that optimized ablation conditions allow resolving 20–40 µm structures without significant blurring artefacts with dimensional deviations typically below 10%. However, these experiments also demonstrate that improving laser spot size alone does not compensate for DGT-driven broadening: DGT-derived images recorded under identical analytical conditions frequently exhibit features broadened by factors of two to three due to pre-ablation lateral diffusion and contact effects. Thus, while high-performance LA-ICP-MS is essential for spatial accuracy, it cannot overcome intrinsic limitations imposed by DGT sampling.

## Conclusions

The findings of this study demonstrate that the effective lateral resolution of DGT LA-ICP-MS imaging is primarily controlled by gel-phase lateral diffusion, gel-sample contact quality, analyte flux and binding kinetics, and the chosen deployment time. When these factors are optimized (i.e., short deployment times, high analyte availability, minimal micro gaps, and the use of a compliant DGT binding gel) practical lateral resolutions of ~ 25 µm can be achieved. Under less favourable conditions, such as extended deployment or irregular contact, the apparent resolution decreases toward the sub-mm scale, consistent with previous observations in environmental applications. Overall, the FWHM-derived lateral diffusion coefficients demonstrate that analyte patterns remain sharply localized, with lateral broadening strongly suppressed by rapid immobilization at the DGT binding phases, yielding apparent *D*_lat_ values several orders of magnitude below molecular diffusion in pure hydrogels. This supports the suitability of the applied PU-C-Zr binding gel for spatially resolved DGT imaging applications, where lateral blurring is an important uncertainty component. Methodologically, we recommend quantifying lateral blur via time-dependent FWHM broadening because it is less sensitive to noise, ROI selection, and low-intensity tails, and thus provides a more transferable and conservative image-resolution metric across elements and measurements. The experimental framework presented here provides a mechanistic basis for interpreting lateral diffusion effects and offers practical guidance for the design of future high-resolution DGT imaging workflows across environmental and materials science.

## Supplementary Information

Below is the link to the electronic supplementary material.

Supplementary file1 (PDF 376 KB)

## Data Availability

The datasets generated during the current study are available from the corresponding author on reasonable request.
