# Supplementary material for: Lateral resolution of DGT LA-ICP-MS for chemical imaging of metal solutes
Source: Anal Bioanal Chem. 2026 Apr 28;418(13):4111–24. doi: 10.1007/s00216-026-06500-7 (PMC13264561; doi:10.1007/s00216-026-06500-7)
Supplement: Supplementary file 1 — Supplementary file1 (PDF 376 KB) [file 216_2026_6500_MOESM1_ESM.pdf]

Supplementary Information

Lateral resolution of DGT LA-ICP-MS for chemical imaging of metal solutes

Gulnaz Mukhametzianova[1,2], Stefan Wagner[1*], Thomas Prohaska[1]

[1]Montanuniversität Leoben, Department of General, Analytical and Physical Chemistry, Chair of General and Analytical Chemistry, Franz-Josef-Strasse 18, 8700 Leoben, Austria

[2]Montanuniversität Leoben, Christian Doppler Laboratory for Inclusion Metallurgy in Advanced Steelmaking, Franz-Josef-Strasse 18, 8700 Leoben, Austria

*Corresponding author: stefan.wagner@unileoben.ac.at; Tel: +43 3842 402 1227

Contents **Page**

**Table S1.** Curated overview of DGT LA-ICP-MS studies (non-exhaustive)	S-2
**Table S2.** Laser ablation settings for PTFE foil patterning	S-4
**Table S3.** Experimental configurations and LA-ICP-MS operating conditions	S-5
**Figure S1.** Photographs of experimental setups	S-6
**Figure S2.** ^{13}C signal intensities for DGT gels analyzed at 50 µm and 5 µm laser spot sizes	S-7
**References**	S-8

Table S1. Curated overview of DGT LA-ICP-MS studies (non-exhaustive), detailing DGT gel binding phases, LA and ICP-MS instrumentation, reported resolutions, and application fields

DGT gel binding phase	LA system	ICP-MS	Resolution	Application field	Reference
Chelex-100, Titanium dioxide, zirconium hydroxide	NWR213, ESL, USA and NWR193, ESI NWR, USA	Agilent 8800, Agilent Technologies and NexION 2000B, PerkinElmer	sub-100 µm	Materials science	[1]
Suspended particulate reagent-iminodiacetate (SPR-IDA)	NWR213, ESI, MT, USA	Agilent 8800, Agilent Technologies, Santa Clara, CA, USA	sub-100 µm	Materials science	[2]
SPR-IDA	UP 193-FX208, ESI, NWR, CA, USA	NexION 300D	not specified	Environmental science	[3]
SPR-IDA, zirconium hydroxide	UP 193-FX, ESI, NWR, Portland, USA	ELAN 9000 DRCe, Perkin-Elmer, Waltham, MA	sub-100 µm	Environmental science	[4]
Zr oxide, AgI, SPR-IDA	UP 193-FX, ESI, NWR	NexION 350D, Perkin Elmer	sub-mm	Environmental science	[5]
SPR-IDA, zirconium hydroxide	UP 193-FX, ESI, NWR, USA	NexION 300D, Perkin Elmer, USA	sub-mm	Environmental science	[6]
SPR-IDA, zirconium hydroxide	UP 193-FX, ESI, NWR Division, Portland OR, USA	NexION 300, Perkin-Elmer	sub-100 µm	Environmental science	[7]
AgI, SPR-IDA	ESI 193FX LA system	Thermo Elemental X-SeriesII	sub-100 µm	Environmental science	[8]
Zirconia	Nd:YAG 213 nm, NWR, USA	Agilent Technologies 7500, USA	sub-mm	Environmental science	[9]
SPR-IDA	UP 193-FX, ESI, NWR, Portland, USA	ELAN 9000 DRCe, Perkin-Elmer, Waltham, MA	sub-mm	Environmental science	[10]
SPR-IDA, zirconium hydroxide, resin DOWEX	NWR 193-FX, ESI, NWR, Portland, USA	NexION 350D	sub-mm	Environmental science	[11]
SPR-IDA, zirconium hydroxide	UP 193-FX, ESI, NWR, Portland, USA	ELAN 9000 DRCe, Perkin-Elmer, Waltham, MA	sub-100 µm	Environmental science	[12]
SPR-IDA, zirconium hydroxide	UP 193-FX, ESI, NWR, Portland, USA	Elan DRCe, Perkin Elmer, Waltham, MA, USA	sub-mm	Environmental science	[13]
Zirconium hydroxide, SPR-IDA	UP 193-FX, ESI, NWR, CA, USA	NexION 300, Perkin-Elmer MA, USA	sub-mm	Environmental science	[14]
SPR-IDA, zirconium hydroxide	UP 193-FX, ESI, NWR, CA, USA	NexION 350, Perkin-Elmer, USA	sub-100 µm	Environmental science	[15]
Titanium dioxide	Not specified	Not specified	sub-mm	Environmental science	[16]
Chelex-100 resin and zirconium hydroxide	NWR imagegeo193, elemental scientific, USA	icpTOF R, Tofwerk, Switzerland	sub-mm	Environmental science	[17]

Table S1. (continued)

DGT gel binding phase	LA system	ICP-MS	Resolution	Application field	Reference
AgI, SPR-IDA	Cetac LSX100	Varian Ultramass ICP-MS	sub-mm	Environmental science	[18]
Chelex-100 resin, zirconium oxide and silver iodide	ASI RESOLution-LRS155 laser microprobe 193 nm ArF laser	Agilent Technologies 7700, USA	sub-mm	Environmental science	[19]
3-mercaptopropyl-trimethoxysilane	Resolution LR/S155 laser ablation system equipped with Coherent Compex-Pro 193 nm ArF excimer laser	Agilent Technologies 7700, USA	sub-mm	Environmental science	[20]
Ferrihydrite	UP-193, New Wave Research, Fremont, CA	ELAN DRCe, Perkin-Elmer, Waltham, MA	sub-mm	Environmental science	[21]
Ferrihydrite	UP-193, New Wave Research, Fremont, CA	ELAN DRCe, Perkin-Elmer, Waltham, MA	sub-100 μm	Environmental science	[22]
Chelex-100	NWR 193, ESI, NWR, Portland, USA	Element XR, Thermo Fisher Scientific, Bremen, Germany	sub-mm	Environmental science	[23]
SPR-IDA	NewWave UP-213	Thermo Electron Series (Hemel Hempstead)	sub-mm	Environmental science	[24]
Silver iodide, iron oxide	New Wave UP-213	Thermo Electron X Series, Hemel Hempstead, UK	sub-mm	Environmental science	[25]
Silver iodide, ferrihydrite	Not specified	Not specified	sub-mm	Environmental science	[26]
SPR-IDA	UP 193-FX, ESI, NWR, CA, USA	NexION 350D, Perkin-Elmer, MA, USA	sub-100 μm	Environmental science	[27]
Chelex-100	NWR213, ESI, Fremont, CA, USA	Agilent Technologies 7700cs, USA	sub-mm	Environmental science	[28]
SPR-IDA, zirconium hydroxide,3-mercaptopropyl-functionalized silica gel	NWR193, ESI, NWR, Portland, OR, USA	NexION 350D, Perkin Elmer, Waltham, MA, USA	sub-mm	Environmental science	[29]
SPR-IDA, zirconium hydroxide	NWR193, ESI, USA	NexION 2000B	sub-mm	Environmental science	[30]
SPR-IDA	Nd:YAG 266 nm CETAC LSX-100	Thermo X7	sub-100 μm	Environmental science	[31]
SPR-IDA	Nd:YAG 213 nm, NWR, Cambridge, U.K.	Thermo X series 2	sub-mm	Environmental science	[32]
SPR-IDA	NWR193, ESI, USA	ELEMENT2, Finnigan, Thermo Electron Corporation, Germany	sub-mm	Environmental science	[33]
Chelex-100, SPR-IDA	ESI 193FX	Thermo Elemental X-Series II	sub-100 μm	Environmental science	[34]

Table S2. Laser ablation settings for PTFE foil patterning

Parameter	Setting
Laser	NWR193 (ESI)
Ablation mode	Burst
Aperture	IVA
Burst counts	1
Passes	25
Carrier gas (He) / L min^{-1}	0.8
Spot size / µm	100, 50, 25, 10, 5
Energy / %	100

Table S3. Experimental configurations and LA-ICP-MS operating conditions

Parameter	Experimental conditions (by experiment/sub-experiment)																
**Experiment**	1		2										3				
Sub-experiment	1.1	1.2	2.1	2.2		2.3	2.4			2.5		2.6	3.1		3.2		
Contact material	PTFE foil		G400PB			Cu TVM							PCB 1		PCB 2		
DGT gel	PU-C-Zr		-	PU-C-Zr		-	PU-C-Zr						PU-C-Zr				
DGT contact time	6 h	24 h	-	15 min		-	15 min			5 min		20 min	15 min				
ROI order	1-15	1-15	1	2	3	4	5	6	7	8	9	10	1	2	3	4	5
Analysis time / h	2.9	2.6	0.2	1.9	2	0.4	1.8	1.4	1.9	0.6	0.6	2.4	4.7	1.3	1.9	1.9	1.9
**Laser ablation system**	imageGEO 193 nm ArF (Elemental Scientific Lasers)																
Ablation mode	Imaging																
Aperture	XYR																
Carrier gas (He) / L min^{-1}	0.8																
Spot size / µm	50		5														
Overlap / µm	45		4														
Scan speed / µm s^{-1}	500		10														
Repetition rate / Hz	100		10														
Energy / J cm^{-2}	6		1	2		1	2						2				
Line length / µm	4000		500	1500		500	1500			500		1500	1500	1000	1500		
Interline distance / µm	50		5														
**ICP-MS**	NexION 2000 (Perkin Elmer)																
Sampler/skimmer cone	Ni																
Plasma gas (Ar) / L min^{-1}	17																
Measured isotopes	^{13}C, ^{27}Al, ^{63}Cu, ^{65}Cu, ^{66}Zn		^{13}C, ^{63}Cu, ^{65}Cu														
Integration time per *m/z* / s	0.01 – 0.02		0.03														
Total acquisition time / s	0.091																

Figure S1. Photographs of experimental setups

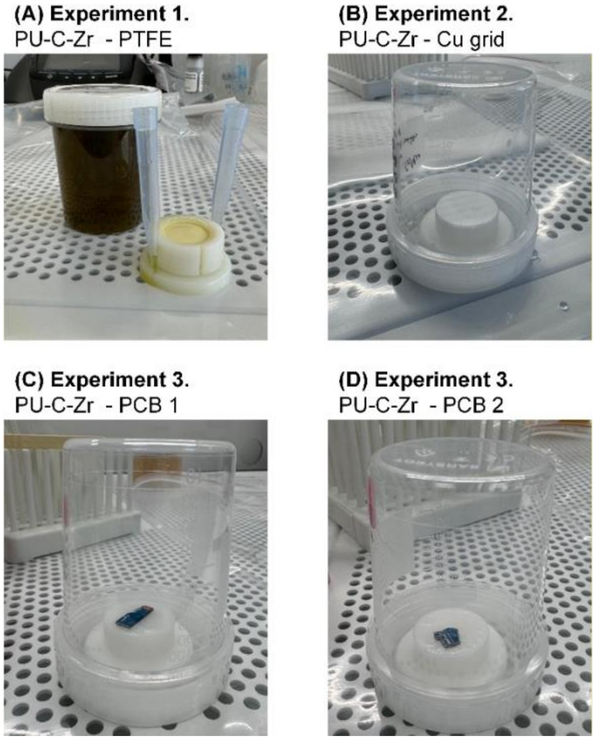

Fig. S1 Photographs of the DGT-based experimental setups used in this study. (A) Piston-type DGT-PTFE immersion setup (with DGT sampler retrieved from immersion solution after sampling), (B) DGT-Cu grid contact setup, (C) DGT-PCB 1, and (D) DGT-PCB 2 contact setup during sampling.

Figure S2. ^{13}C intensities for DGT gels analyzed at 50 µm and 5 µm laser spot sizes

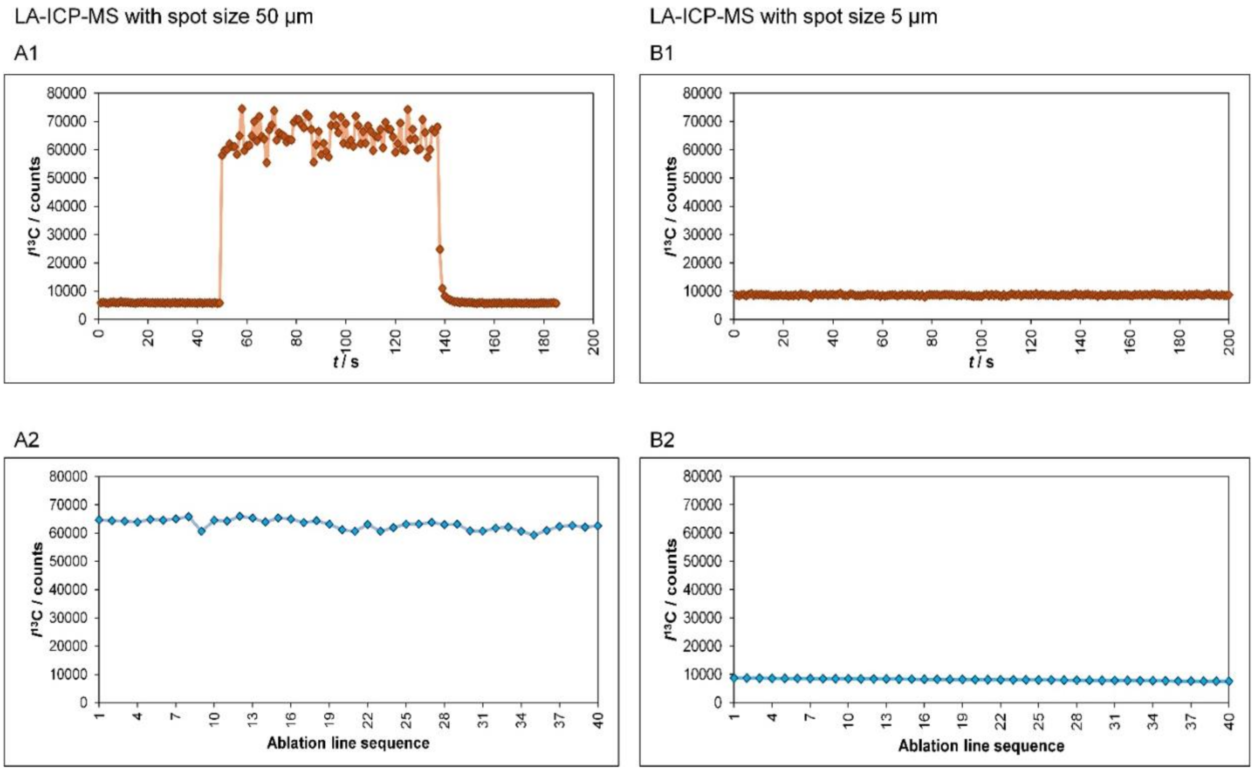

Fig. S2 ^{13}C signal behavior during LA-ICP-MS ablation of DGT gels at different laser spot sizes. (A) PU-C-Zr gel ablated with a 50 µm spot size. (B) PU-C-Zr gel ablated with a 5 µm spot size. Subpanels A1 and B1 show the ^{13}C intensity along the first ablation line, whereas A2 and B2 show the average ^{13}C intensity per line over 40 consecutive ablation lines

References

1. Mukhametzianova G, Wagner S, Eskinja M, Moshtaghi M, Mori G, Prohaska T. Mapping elemental solutes at sub-picogram levels during aqueous corrosion of Al alloys using diffusive gradients in thin films (DGT) with LA-ICP-MS. Analytical and Bioanalytical Chemistry 2024; 416: 3373–3388. https://doi.org/10.1007/s00216-024-05288-8.

2. Wagner S, Hummel C, Santner J, Puschenreiter M, Irrgeher J, Wenzel WW, Borisov SM, Prohaska T. In situ spatiotemporal solute imaging of metal corrosion on the example of magnesium. Analytica Chimica Acta 2022; 1212: 339910. https://doi.org/10.1016/j.aca.2022.339910.

3. Álvarez-López V, Puschenreiter M, Santner J, Lehto N, Prieto-Fernández Á, Wenzel WW, Monterroso C, Kidd PS. Evidence for nickel mobilisation in rhizosphere soils of Ni hyperaccumulator Odontarrhena serpyllifolia. Plant and Soil 2021; 464: 89–107. https://doi.org/10.1007/s11104-021-04944-7.

4. Bilyera N, Hummel C, Daudin G, Santangeli M, Zhang X, Santner J, Lippold E, Schlüter S, Bertrand I, Wenzel W, Spielvogel S, Vetterlein D, Razavi BS, Oburger E. Co-localised phosphorus mobilization processes in the rhizosphere of field-grown maize jointly contribute to plant nutrition. Soil Biology and Biochemistry 2022; 165: 108497. https://doi.org/10.1016/j.soilbio.2021.108497.

5. Brodersen KE, Koren K, Moßhammer M, Ralph PJ, Kühl M, Santner J. Seagrass-Mediated Phosphorus and Iron Solubilization in Tropical Sediments. Environmental Science & Technology 2017; 51: 14155–14163. https://doi.org/10.1021/acs.est.7b03878.

6. Christel W, Zhu K, Hoefer C, Kreuzeder A, Santner J, Bruun S, Magid J, Jensen LS. Spatiotemporal dynamics of phosphorus release, oxygen consumption and greenhouse gas emissions after localised soil amendment with organic fertilisers. Science of The Total Environment 2016; 554-555: 119–129. https://doi.org/10.1016/j.scitotenv.2016.02.152.

7. Fresno T, Peñalosa JM, Santner J, Puschenreiter M, Moreno-Jiménez E. Effect of Lupinus albus L. root activities on As and Cu mobility after addition of iron-based soil amendments. Chemosphere 2017; 182: 373–381. https://doi.org/10.1016/j.chemosphere.2017.05.034.

8. Gao Y, van de Velde S, Williams PN, Baeyens W, Zhang H. Two-dimensional images of dissolved sulfide and metals in anoxic sediments by a novel diffusive gradients in thin film probe and optical scanning techniques. TrAC Trends in Analytical Chemistry 2015; 66: 63–71. https://doi.org/10.1016/j.trac.2014.11.012.

9. Guan D-X, Williams PN, Luo J, Zheng J-L, Xu H-C, Cai C, Ma LQ. Novel Precipitated Zirconia-Based DGT Technique for High-Resolution Imaging of Oxyanions in Waters and Sediments. Environmental Science & Technology 2015; 49: 3653–3661. https://doi.org/10.1021/es505424m.

10. Hoefer C, Santner J, Puschenreiter M, Wenzel WW. Localized Metal Solubilization in the Rhizosphere of Salix smithiana upon Sulfur Application. Environmental Science & Technology 2015; 49: 4522–4529. https://doi.org/10.1021/es505758j.

11. Hoefer C, Santner J, Borisov SM, Wenzel WW, Puschenreiter M. Integrating chemical imaging of cationic trace metal solutes and pH into a single hydrogel layer. Analytica Chimica Acta 2017; 950: 88–97. https://doi.org/10.1016/j.aca.2016.11.004.

12. Hummel C, Boitt G, Santner J, Lehto NJ, Condron L, Wenzel WW. Co-occurring increased phosphatase activity and labile P depletion in the rhizosphere of Lupinus angustifolius assessed with a novel, combined 2D-imaging approach. Soil Biology and Biochemistry 2021; 153: 107963. https://doi.org/10.1016/j.soilbio.2020.107963.

13. Hummel C, Daudin G, Gerzabek MH, Santner J, Wenzel WW, Oburger E. Chemical imaging reveals environmental risk of minor tungsten and lead shotgun pellet constituents during weathering in soil. Science of The Total Environment 2023; 882: 163554. https://doi.org/10.1016/j.scitotenv.2023.163554.

14. Kreuzeder A, Santner J, Prohaska T, Wenzel WW. Gel for Simultaneous Chemical Imaging of Anionic and Cationic Solutes Using Diffusive Gradients in Thin Films. Analytical Chemistry 2013; 85: 12028–12036. https://doi.org/10.1021/ac403050f.

15. Kreuzeder A, Santner J, Scharsching V, Oburger E, Hoefer C, Hann S, Wenzel WW. In situ observation of localized, sub-mm scale changes of phosphorus biogeochemistry in the rhizosphere. Plant and Soil 2018; 424: 573–589. https://doi.org/10.1007/s11104-017-3542-0.

16. Li X, Zuo J, Zhang C, Zhang T, He Z, Zhou Q, Zhao Y, Liu W. High-resolution two-dimensional mapping of arsenic concentration in soil-water micro-interfaces with diffusive gradients in thin films. Environmental Chemistry and Ecotoxicology 2025; 7: 211–220. https://doi.org/10.1016/j.enceco.2024.12.006.

17. Liu C, Ding T-X, van der Ent A, Liu C, Morel JL, Sirguey C, Liu W-S, Tang Y-T, Qiu R-L. A novel method for in situ imaging of root exudates and labile elements reveals phosphorus deficiency-induced mobilization of rare earth elements in the rhizosphere of Phytolacca americana. Plant and Soil 2024; 495: 13–26. https://doi.org/10.1007/s11104-023-06146-9.

18. Motelica-Heino M, Naylor C, Zhang H, Davison W. Simultaneous Release of Metals and Sulfide in Lacustrine Sediment. Environmental Science & Technology 2003; 37: 4374–4381. https://doi.org/10.1021/es030035+.

19. Ren M, Ding S, Dai Z, Wang J, Li C, Zhong Z, Cao J, Yang L, Tsang DCW, Xu S, Yang C, Wang Y. A new DGT technique comprising a hybrid sensor for the simultaneous high resolution 2-D imaging of sulfides, metallic cations, oxyanions and dissolved oxygen. Journal of Hazardous Materials 2021; 403: 123597. https://doi.org/10.1016/j.jhazmat.2020.123597.

20. Ren M, Zhong Z, Ding S, Wang J, Dai Z, Li C, Cao J, Wang Y, Yu Z, Zhang C. Selective and simultaneous high resolution 2-D imaging of AsIII, CrIII and SbIII and dissolved oxygen by developing a new DGT technique comprising a hybrid sensor. Science of The Total Environment 2022; 835: 155460. https://doi.org/10.1016/j.scitotenv.2022.155460.

21. Santner J, Prohaska T, Luo J, Zhang H. Ferrihydrite Containing Gel for Chemical Imaging of Labile Phosphate Species in Sediments and Soils Using Diffusive Gradients in Thin Films. Analytical Chemistry 2010; 82: 7668–7674. https://doi.org/10.1021/ac101450j.

22. Santner J, Zhang H, Leitner D, Schnepf A, Prohaska T, Puschenreiter M, Wenzel WW. High-resolution chemical imaging of labile phosphorus in the rhizosphere of Brassica napus L. cultivars. Environmental and Experimental Botany 2012; 77: 219–226. https://doi.org/10.1016/j.envexpbot.2011.11.026.

23. Smolders E, Wagner S, Prohaska T, Irrgeher J, Santner J. Sub-millimeter distribution of labile trace element fluxes in the rhizosphere explains differential effects of soil liming on cadmium and zinc uptake in maize. Science of The Total Environment 2020; 738: 140311. https://doi.org/10.1016/j.scitotenv.2020.140311.

24. Stahl H, Warnken KW, Sochaczewski L, Glud RN, Davison W, Zhang H. A combined sensor for simultaneous high resolution 2-D imaging of oxygen and trace metals fluxes. Limnology and Oceanography: Methods 2012; 10: 389–401. https://doi.org/10.4319/lom.2012.10.389.

25. Stockdale A, Davison W, Zhang H. High-resolution two-dimensional quantitative analysis of phosphorus, vanadium and arsenic, and qualitative analysis of sulfide, in a freshwater sediment. Environmental Chemistry 2008; 5: 143. https://doi.org/10.1071/EN07096.

26. Stockdale A, Davison W, Zhang H. 2D simultaneous measurement of the oxyanions of P, V, As, Mo, Sb, W and U. Journal of Environmental Monitoring 2010; 12: 981–984. https://doi.org/10.1039/B925627J.

27. Valentinuzzi F, Mimmo T, Cesco S, Al Mamun S, Santner J, Hoefer C, Oburger E, Robinson B, Lehto N. The effect of lime on the rhizosphere processes and elemental uptake of white lupin. Environmental and Experimental Botany 2015; 118: 85–94. https://doi.org/10.1016/j.envexpbot.2015.06.010.

28. Vanderschueren R, Doevenspeck J, Helsen F, Mounicou S, Santner J, Delcour JA, Chavez E, Smolders E. Cadmium migration from nib to testa during cacao fermentation is driven by nib acidification. LWT - Food Science and Technology 2022; 157: 113077. https://doi.org/10.1016/j.lwt.2022.113077.

29. Wagner S, Hoefer C, Puschenreiter M, Wenzel WW, Oburger E, Hann S, Robinson B, Kretzschmar R, Santner J. Arsenic redox transformations and cycling in the rhizosphere of Pteris vittata and Pteris quadriaurita. Environmental and Experimental Botany 2020; 177: 104122. https://doi.org/10.1016/j.envexpbot.2020.104122.

30. Wagner S, Hoefer C, Prohaska T, Santner J. Two-Dimensional Visualization and Quantification of Labile, Inorganic Plant Nutrients and Contaminants in Soil. Journal of Visualized Experiments 2020; 163: e61661. https://doi.org/10.3791/61661.

31. Warnken KW, Zhang H, Davison W. Analysis of Polyacrylamide Gels for Trace Metals Using Diffusive Gradients in Thin Films and Laser Ablation Inductively Coupled Plasma Mass Spectrometry. Analytical Chemistry 2004; 76: 6077–6084. https://doi.org/10.1021/ac0400358.

32. Williams PN, Santner J, Larsen M, Lehto NJ, Oburger E, Wenzel W, Glud RN, Davison W, Zhang H. Localized Flux Maxima of Arsenic, Lead, and Iron around Root Apices in Flooded Lowland Rice. Environmental Science & Technology 2014; 48: 8498–8506. https://doi.org/10.1021/es501127k.

33. Wu Z, Wang S, Ji N. Distribution character of localized iron microniche in lake sediment microzone revealed by chemical image. Environmental Science and Pollution Research International 2019; 26: 35704–35716. https://doi.org/10.1007/s11356-019-06219-2.

34. Zhou C, van de Velde S, Baeyens W, Gao Y. Comparison of Chelex based resins in diffusive gradients in thin-film for high resolution assessment of metals. Talanta 2018; 186: 397–405. https://doi.org/10.1016/j.talanta.2018.04.085.
